# Impacts of early-life thermal challenge on development, survival, and physiology in wild great tits

**DOI:** 10.1242/bio.062670

**Published:** 2026-09-11

**Authors:** Julie Fleitz, Clémence Furic, Charli S. Davies, Jenna Palttala, Valentina Giongo, Loan Hognon, Suvi Ruuskanen, Sophie Reichert

**Affiliations:** ^1^ Department of Biology, University of Turku, 20500 Turku, Finland; ^2^ Department of Ecology, Physiology and Ethology, Université de Strasbourg, CNRS, IPHC UMR 7178, 67000 Strasbourg, France; ^3^ Department of Biological and Environmental Science, University of Jyväskylä, 40014 Jyväskylä, Finland; ^4^ Department of Biological, University of Padua, 35122 Padova, Italy

**Keywords:** Climate change, Ecology, Heat wave, Cold snap, Nestling, Life history, Passerine bird

## Abstract

Rapid climate change is increasing thermal extremes that may challenge developing endotherms. In altricial birds, early postnatal life is a sensitive period because thermoregulatory and physiological systems are still maturing. We experimentally manipulated nest temperature in a wild population of great tit to test whether mild, ecologically realistic heat (+2.3°C) and cold (−1.3°C) challenges affected development and physiology. Treatments were applied from days 2-8 post-hatching, and we measured growth, survival, hydration status (plasma osmolarity and uric acid), mitochondrial density, and immunoglobulin levels. Contrary to our predictions, thermal manipulation had no detectable effects on survival, morphology, or physiological traits at either day 8 or day 14. In contrast, all physiological markers varied significantly with age, reflecting normal osmoregulatory, metabolic, and immune development. These findings suggest that moderate, short-term thermal deviations during early development fall within the tolerance range of this population, or that parental behaviour buffered nestlings against temperature changes. However, stronger or prolonged extremes may exceed buffering capacity, and delayed effects cannot be excluded. Long-term studies are needed to assess latent fitness costs under increasingly variable climates.

## Teaser

**Summary:** Experimental warming and cooling of nests revealed that young birds can tolerate moderate short-term temperature fluctuations during early development.

## INTRODUCTION

Rapid climate change is exposing wildlife to increasing climatic variability, including more frequent, long, and intense heatwaves and cold snaps (IPCC, 2023; Fischer et al., 2021). Such extreme, stochastic and short-term weather events can impose strong selective pressures on endotherms by altering energy balance, thermoregulatory demands, and ultimately, survival and reproduction (McKechnie and Wolf, 2019; Lv et al., 2023; Wolfe et al., 2025). Importantly, when thermal challenge occurs during sensitive developmental phases, they may have effects that extend well beyond the period of exposure (Hepp et al., 2015; Nord and Giroud, 2020).

Early postnatal development in birds represents one such critical phase. For most passerines, the thermoneutral zone (TNZ) lies approximately between 15 and 35°C (Gavrilov and Dolnik, 1985; Playà-Montmany et al., 2021; Cabello-vergel et al., 2022). However, this concept does not translate directly to early altricial nestlings, which have not yet achieved endothermic competence and are largely dependent on ambient nest environment (i.e. parental brooding and nest microclimate) for thermoregulation (Price and Dzialowski, 2018; Katzenberger et al., 2015; Boyles et al., 2011; Dawson and O'Connor, 1996). Previous studies in endothermic species have experimentally altered postnatal temperatures in the wild, revealing effects on growth and thermoregulation (Dawson et al., 2005; Rodríguez and Barba, 2016a,b; Nord and Giroud, 2020; Stier et al., 2021; Weeks et al., 2022; Casagrande and Dell'Omo, 2025; Woodruff et al., 2025). For example, an experimental study on great tit (*Parus major*, Linnaeus, 1758) populations found that increased nest temperatures during the nestling stage impaired growth, with nestlings from heated nests having lower mass than controls by day 15 – just before fledging (Rodríguez and Barba, 2016b). Similarly, experimentally increased nest temperatures in blue tit (*Cyanistes caeruleus,* Linnaeus, 1758) nestlings raised their body temperatures, and reduced their body mass gain relative to controls (Andreasson et al., 2018).

The physiological mechanisms underlying thermal challenge are relatively well characterized, particularly in controlled settings. Heat and cold exposure affect several key biological systems including mitochondrial function, reducing the efficiency of ATP production while often stimulating mitochondrial proliferation (Akbarian et al., 2016), and immune function, frequently leading to a reduction in antibody production and immune cell activity (Siddiqui et al., 2022; Li et al., 2025). However, studies in wild bird show that these responses are often strongly context-dependent and can vary with environmental conditions, developmental stage, and parental buffering (Taff et al., 2026; Taff and Shipley, 2023). Additionally, responses to thermal variation may also depend on population history and local adaptation. In great tits, population differences in reaction norms to environmental conditions have been reported even in the presence of gene flow (Broggi et al., 2005), indicating that phenotypic plasticity can vary across populations. This suggests that responses to thermal variation may not be uniform across the species' range, highlighting the value of quantifying developmental responses in ecological realistic conditions within specific populations.

In this study, we experimentally manipulated nest temperature during early postnatal development in wild great tits to test how heat and cold challenges affect nestling growth, survival, and physiological condition. The magnitude of our temperature manipulation (±2°C) was chosen to reflect near-term climate change projections (IPCC, 2023), representing the chronic thermal deviation nestlings are likely to experience within coming decades, as opposed to the acute extremes that characterize current heatwaves or cold snaps. While such deviations are modest relative to the natural daily temperature fluctuations, it is precisely this ecologically realistic scale that is most relevant for predicting population-level responses to ongoing climate change.

Temperature treatments were applied from two to eight days post-hatching (d2-d8), when nestlings only begin to develop endothermic capacity (Dawson and O'Connor, 1996), therefore covering the period of highest sensitivity to external temperature variation. We measured body mass, wing length, and survival across development as well as physiological responses including hydration status, immune function (immunoglobulin levels), and mitochondrial density as these markers reflect key pathways through which thermal challenge affects growth, water balance, immunity, and metabolic capacity.

To our knowledge, relatively few studies have simultaneously assessed both developmental and physiological responses in the wild, and most have focused on temperature manipulations during later nestling stages, when thermoregulatory capacity is already emerging i.e. when nestlings are beginning to achieve endothermic competence and are less dependent on parental brooding and nest microclimate for maintaining their body temperature. Because maintaining body temperature under either heat or cold challenge during this vulnerable developmental period can divert energy away from growth processes, we predicted that nestlings in both heated and cooled nests would exhibit (i) reduced body mass and wing length compared with controls, (ii) increased mortality risk during the nestling period due to heightened energetic demands, dehydration, or thermoregulatory failure, (iii) signs of dehydration in heat-exposed nestlings (elevated plasma osmolarity and uric acid), (iv) increased mitochondrial density (as a compensatory response to increased energy demand) in both heat and cold groups and (v) reduced plasma immunoglobulin concentrations through stress-induced immune suppression.

## MATERIALS AND METHODS

### Ethical consideration and health monitoring

All procedures were approved by the Animal Experiment Committee of the State Provincial Office of Southern Finland (license no. ESAVI/8878/2024) and by the Centre of Economic Development Transport and the Environment (VARELY/1075/2024). Bird ringing permits were assigned by the National Ringing Center under Helsinki Natural History Museum.

### Experimental design

The experiment was carried out in a nest box population (comprising five study sites within 10-km distance) of wild great tits around Jyväskylä, Finland (62.25°N, 25.78°E). Great tits offer an ideal model to study developmental responses to thermal variability as it is a widespread, well-studied passerine that breeds in nest boxes, allowing precise experimental manipulation of the nest microclimate and repeated measurements of nestlings (Corregidor-Castro and Jones, 2021; Rodríguez and Barba, 2016a,b). In high-latitude populations such as those in Finland, nestlings are naturally exposed to pronounced short-term temperature variability during the breeding season (Nord and Nilsson, 2016), making them a relevant system for studying developmental responses to thermal variation under ecologically realistic conditions in the wild. In great tits, clutch size varies from 7 to 12 eggs (Perrins and McCleery, 1989), only females incubate for the first 13 to 14 days post hatching with the nestling period lasting from 16 to 22 days. After hatching, both sexes participate in feeding the young (Álvarez and Barba, 2014).

At the onset of the 2024 breeding season, nest boxes were inspected weekly to record initial species occupancy [great tits, blue tits, and pied flycatchers (*Ficedula hypoleuca,* Pallas, 1764)], egg-laying date, clutch size (between 5 and 10 eggs), and onset of incubation. Eggs were laid between 1st and 17th May, and nestlings hatched between 21st May and 6th June, ensuring a broad temporal distribution across the breeding season. These inspections allowed hatching dates to be estimated with ±24 h accuracy.

Nest boxes used by great tits and included in the experiment (*n*=57) were assigned to one of the control (*n*=18; 136 nestlings), heat (*n*=20; 144 nestlings) or cold (*n*=19; 139 nestlings) treatment groups. Treatments were applied randomly but balanced by location, hatching date, and brood size (average: 7.7 nestlings, range: 2-10). Treatment consisted on the installation of two heating pads (AquaPack HeatPaxx 72 h) or cooling pads (VWR 606 g, frozen at −20°C) in pouches on the outer lateral sides of the wooden nest boxes, surrounded by insulation, to modify nest temperature. This resulted in an average increase of approximately +2.3°C in heated nests and a decrease of approximately −1.3°C in cooled nests (Stier et al., 2021; Furic et al., 2026). Pads were replaced every two days, between 07:00-17:30. Control nests received inactive pads with insulation, and were also visited every second day to standardize human disturbance across treatments. Nest temperature was monitored using thermo-loggers (iButton® DS1923 temperature/humidity logger,±0.06°C and 0.04%RH Resolution) placed inside the nest box at the beginning of the treatment (d2), 5 cm above the chicks, measuring temperature every 10 min. Therefore, temperature recorded reflects the actual nest microclimate experienced by nestlings, which is shaped by ambient conditions and parental brooding behaviour (Álvarez and Barba, 2014). Additionally, two to three thermo-loggers (EL-USB-2 RH/TEMP USB data logger) were evenly distributed at each site and installed on the outside part of unoccupied nest boxes in order to measure the local ambient humidity and temperature.

### Individual identification and sampling

For longitudinal tracking, nestlings were first marked at two days old using the nail-clipping method, and then ringed at eight days old with uniquely numbered metal rings on the right tarsus. Nestlings were weighed at 2 (pre-treatment), 8 (during the treatment), and 14 days old (post-treatment), body mass was recorded to the nearest 0.01 g with a digital balance. Wing length was measured at 8 and 14 days old to the nearest 0.1 mm with a wing ruler. Nestling survival was monitored at 2, 4, 6, 8, and 14 days old. As some nestlings were transferred to captivity at day 14 alongside their parents for a separate experiment, survival monitoring was discontinued at this age to avoid confounding natural survival with the effects of further experimental manipulations.

### Blood sampling

Blood was collected at 8 (∼30 μl) and 14 (∼60 μl) days old. Samples were drawn from the brachial vein using heparinized capillaries and stored in Eppendorf tubes. Whole blood was kept on ice until centrifugation at the end of the day, after which plasma and red blood cells were separated and frozen at −80°C (±5 months before analysis).

### Plasma immunoglobulin assay (ELISA)

Total plasma immunoglobulins (IgG) were quantified as an immune function marker (Tizard, 2024) using an established ELISA protocol (Ruuskanen et al., 2015, n_control_=158, n_heat_=193, n_cold_=180). We diluted 2.5 μl of pure plasma in 1% BSA-PBS with a final dilution at 1:5000, run in triplicate on 96-well plates. Absorbance was read at 405 nm (Tecan Spark). A pooled plasma standard (500,000 U/ml) was serially diluted (10,000–0 U/ml) to generate a four-parameter logistic calibration curve, included on each plate. IgG concentrations were interpolated from the curve, corrected for dilution, and accepted when triplicate CV <10%.

### Plasma dehydration markers

Birds excrete nitrogen mainly as uric acid. When dehydrated, they reduce water loss by excreting more concentrated urates. This often leads to elevated uric acid concentrations in the plasma. To assess the amount of uric acid in plasma, we used the Randox UA230 enzymatic colorimetric kit (Randox Laboratories Ltd, UK). For each sample (n_control_=122, n_heat_=166, n_cold_=133), 2 μl were tested and duplicated if possible. In addition to the calibrator kit, we incorporated two inter-plate controls, a reconstituted lyophilized human plasma and a fresh human plasma. Next, 100 μl of enzyme reagent was added to each well. After incubating the plate at 37°C for 5 min, we read the absorbances at 520 nm. The uric acid concentration was evaluated based on the average of the raw blank and calibrator data multiplied by the standard concentration corresponding to 9.95. Extreme values (CV>30%) were removed from the dataset.

Plasma osmolarity increases when water is lost relative to solutes. Plasma osmolarity was quantified from plasma samples using an osmometer. As plasma quantities were very limited, each sample (n_control_=98, n_heat_=100, n_cold_=97) was diluted with MilliQ water in a 1:4 ratio. To verify the effectiveness of the osmometer over time, we used the same human plasma diluted 1:4 in each session. Extreme values (<250 mosml/l and >400 mosml/l) were removed from the dataset.

### Molecular sexing and mitochondrial density

DNA was extracted from 5 μl red blood cells and eluted to 180 μl following the manufacturer's protocol for animal tissues. DNA was quantified using a Quant-IT PicoGreen dsDNA Assay Kit (Thermo Fisher Scientific), and final concentrations were standardized to 1.2 ng μl in nuclease-free water.

Mitochondrial DNA copy number (mtDNAcn), used as an index of mitochondrial density, was quantified by real-time quantitative PCR (qPCR). The relative mtDNAcn was calculated as the ratio between a mitochondrial gene (cytochrome c oxidase subunit 1, COI) and a nuclear single-copy gene (RAG1), following primer design based on the great tit genome. qPCR reactions were run in triplicate on a QuantStudio 12K Flex system using SensiFAST™ SYBR® Lo-ROX (Bioline) and the following thermal profile: 95°C for 3 min, followed by 40 cycles of 95°C for 10 s, 58°C for 15 s, and 72°C for 10 s. Melt-curve analysis was performed to verify product specificity, and amplification efficiency (80–120%, R^2^ ≥0.99) was calculated from standard curves. Relative mtDNAcn was computed as 2^−^ΔCt, where ΔCt=Ct_COI – Ct_RAG1.

Birds were molecularly sexed at d8 via PCR following Griffiths et al. (1998). Reactions (12 μl) contained 1.5 μl of gDNA, 250 nM of each primer (P2: 5′-TCTGCATCGCTAAATCCTTT-3′; P8: 5′-CTCCCAAGGATGAGRAAYTG-3′), and 6.25 μl of Mytaq HS red mix (Meridian Bioscience), and 4.5 μl RNase/DNA-free molecular grade water. The thermal profile consisted of 94°C for 1:30 min followed by 30 cycles of 58°C for 30 s, 72°C for 45 s, 94°C for 30 s. Reactions were finished with 58°C for 1 min, followed by final elongation of 72°C for 5 min. Products were verified and sexes scored on a 2% agarose gel.

### Parental behavior

To assess whether treatment affects parental care, we video-recorded a subsample of nest boxes (N_Control_=6, N_Heat_=15, N_Cold_=10) 4 days after hatching. Cameras were installed at approximately 2 m distance from the nest boxes. Videos were recorded for approximately 1 h (mean±s.d.=65.97±40.25 min) between 09:00-17:00. Standardized parental feeding rate differences (number of nest visits by both parents divided by the total length of each video) was quantified using BORIS software (Friard and Gamba, 2016).

### Statistical analysis

All analyses were conducted in RStudio (version 2025.09.0; R Core Team, 2024). We used mixed-effects models to evaluate treatment effects on survival, morphological, and physiological variables while accounting for repeated measures and hierarchical data structure. Models were fitted using maximum likelihood (REML=FALSE), and Type III Wald χ^2^ tests (*car* package) were used to assess fixed effects. Post hoc comparisons were performed with *emmeans* (Lenth and Piaskowski, 2026), applying Tukey corrections for multiple testing. Model assumptions (normality, homogeneity and dispersion) were checked using DHARMa (v.0.4.7), and variables were log-transformed when necessary. Figures were produced with *ggplot2* (v.3.5.2) and *ggpubr* (v.0.6.0). Statistical significance was defined as *P*<0.05.

#### Environmental and survival analyses

Treatment effects on nest box temperature and humidity were analyzed using a generalized additive mixed model (GAMM) (mgcv package; Wood, 2017). Temperature or humidity recorded at 10-min intervals between the first and last treatment were used as the response variable. Treatment (Control, Cold, Heat) and d2 brood size were included as a fixed factor while Nest ID and Site were included as random effects. To account for temporal structure in the data, we included a cyclic smooth term for time of day (hour of day expressed on a continuous 0-24 scale) to model the diurnal temperature and humidity cycle. Calendar date was included as a continuous numeric variable to model gradual temporal variation across the experiment. Scaled hatch date was included to account for seasonal variation in breeding timing (i.e. early versus late nests, which differ in day length and potentially availability of insect food), ensuring that developmental outcomes were evaluated independently of seasonal progression. The model assumed a Gaussian error distribution with an identity link.

To evaluate how nest box temperatures varied relative to ambient conditions, we calculated the temperature difference between nest box and concurrent ambient temperature (ΔT=nest box temperature−ambient temperature, using USB logger data) and compared ΔT among treatments (Control, Cold, Heat) using the same GAMM structure described above.

Nestling survival to day 14 was analyzed with a mixed-effects Cox proportional hazards model (*coxme* package; Therneau, 2022), including treatment as a fixed effect and nest box nested within site as a random factor. Since molecular sexing determination required a blood sample on day 8, it was not possible to determine the sex of nestlings that died before that age, therefore, we did not include the sex variable in the survival model. We report hazard ratios (HR) with 95% confidence intervals, and Kaplan–Meier curves were plotted to visualize survival patterns among treatments (Fig. 2).

#### Morphometric and physiological analyses

Treatment effects on morphological (body mass and wing length) and physiological (mitochondrial density, IgG concentration, Uric acid concentration, and plasma osmolarity) traits were tested using linear mixed-effects models (LMMs) (*lme4* package; Bates et al., 2015). Fixed effects included treatment, age (2, 8, 14 days for body mass; 8 and 14 days for wing length and physiology), and their interaction. Age at day 2 refers to pre-treatment, day 8 during treatment, and day 14 post-treatment. Scaled hatch date, d2 brood size, scaled hour of capture, sex (for morphological traits only) and plasma hemolyse (for uric acid concentration only) were included as covariates. Sex was excluded from physiological trait analyses as it did not significantly contribute to the models, allowing us to maximize sample size. Random intercepts for nestling ID, nest box, site and (for physiological traits) plate number, accounted for repeated and nested measurements.

#### Parental behavior

Standardized parental feeding rate differences were tested according to treatment groups using a linear model. Time of the day (h), brood size at day 2 and site were included as covariates. Model residuals met assumptions of normality and equal variance.

## RESULTS

### Nest-box temperature

A total of 47 temperature and humidity datasets were obtained from 57 monitored nests. Data of 4 iButtons were censored as they were either displaced inside the nests or never recovered. Data were included up to the last day all chicks in a nest were recorded alive. Six iButtons were excluded from the final dataset as all chicks in the corresponding nests had died by day 4.

**Fig. 1.**
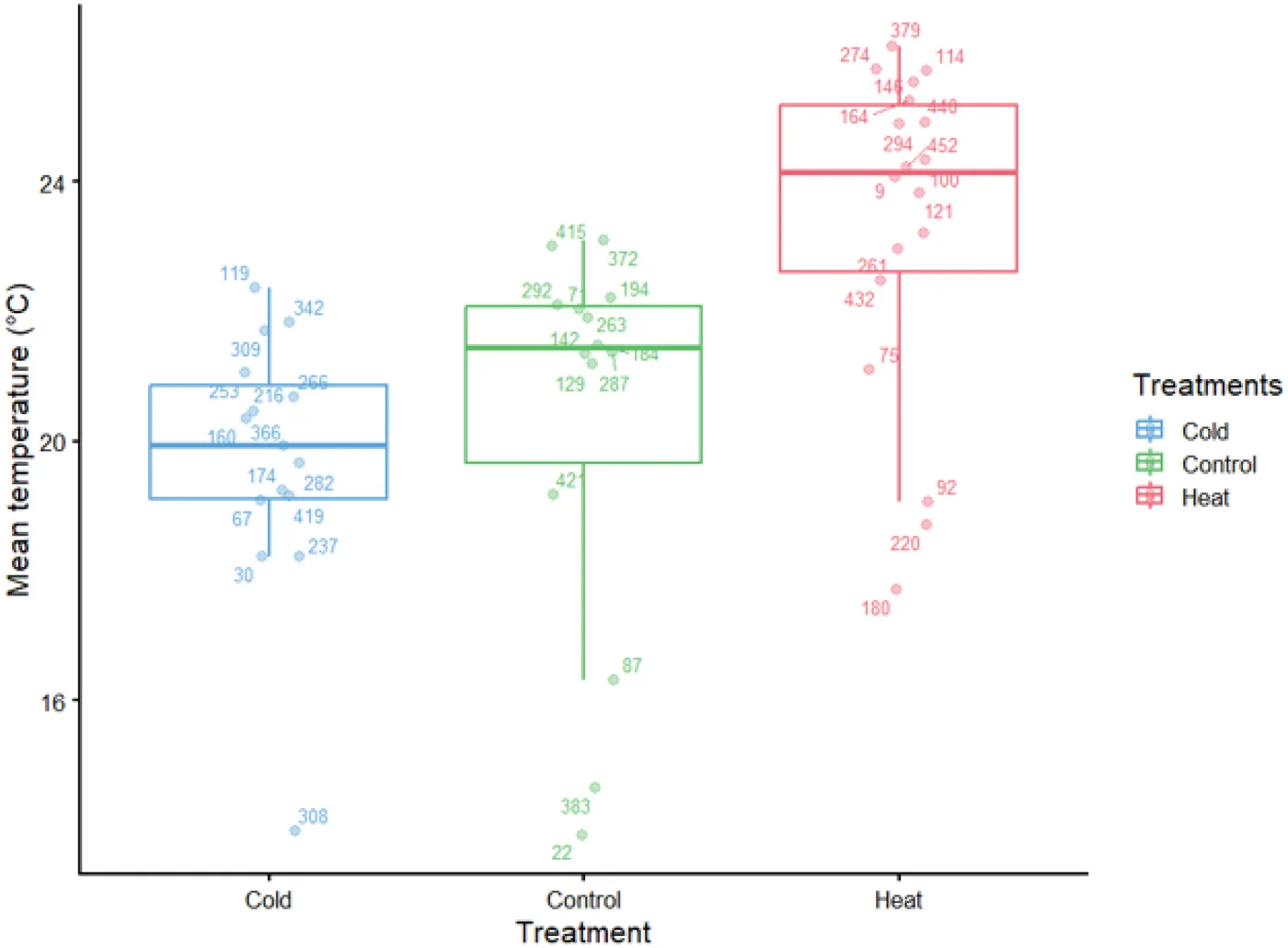
**Nest temperature during early life across thermal treatment groups.** Boxplots show the nest temperature (°C) for Cold, Control, and Heat treatment groups. Boxes represent the interquartile range (IQR), horizontal lines indicate the median and nest boxes data points are shown as jittered dots and overlaid with individual identifiers.

The GAMM model explained a substantial portion of the variance (adjusted R^2^=0.63). Over the entire treatment period (i.e. day 2 to day 8) temperature inside the nest box was significantly altered: Heated boxes were 2.32±0.55°C warmer than the control ones (t=4.22, *P*<0.001), whereas cold boxes were 1.31±0.57°C cooler (t=−2.30, *P*=0.02). Time of day (edf=14.91, F=1447, *P*<0.001) and calendar date (edf=6.99, F=1597, *P*<0.001) also had significant effects, showing strong daily and seasonal fluctuations in temperature (Fig. 1, Fig. S1). The significant effect of calendar date reflects seasonal changes in ambient temperature across the breeding season, which are transmitted into nest boxes, while treatment effects represent deviations from this background temporal variation. Nest temperatures were unrelated to brood size (t=0.98, *P*=0.33). When considering only a short time-window after treatment was applied every second day, i.e., short term temperature variation between 12:00-20:00 when the day is hottest, heated boxes were 1.94±0.55°C warmer than the control ones (t=3.49, *P*<0.001), and cold boxes were 2.02±0.58°C cooler (t=−3.5, *P*<0.001).

**Fig. 2.**
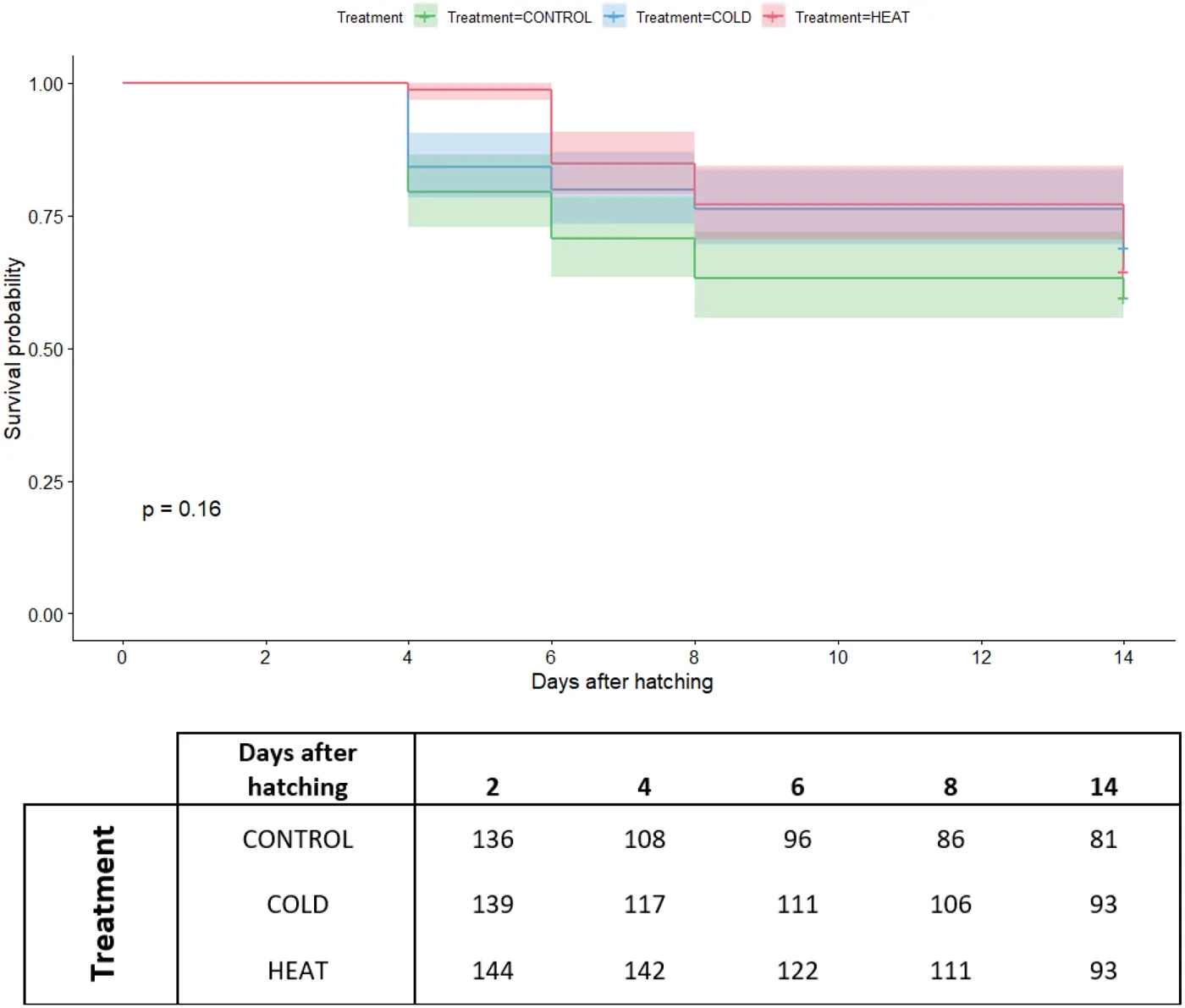
Survival probability with the 95% confidence intervals for the Control, Heat and Cold treatment groups according to time after hatching (in days).

On average, nest box temperature in heat group was 4.5°C (95% CI: 4.4-4.5) above concurrent ambient temperature, cold-treated nests were 0.5°C (95% CI: 0.4-0.5) above ambient, and control nests were 1.1°C (95% CI: 1-1.4) relative to ambient, presumably reflecting the basic insulating effect of the nest box itself as well as heat generated by the nestlings and parents.

Humidity did not differ between cold versus control boxes (estimate=2.75±4.61, *P*=0.55) or heated versus control boxes (estimate =−4.79±4.51, *P*=0.29).

### Nestling survival

Of 419 nestlings monitored, 116 died before d14. Most variation in survival was attributable to differences among nest boxes (random intercept s.d.=2.39), with smaller variation among areas (s.d.=0.1). Treatment had no significant effect on survival to d14 (Cold: HR=−0.57, 95% CI 0.56-0.99, *P*=0.57; Heat: HR=−0.69, 95% CI 0.50-0.92, *P*=0.452, Fig. 2).

### Morphometrics

Age, brood size, and the treatment×age interaction significantly affected log-transformed chick mass (Age: χ^2^=4392.37, d.f.=2, *P*<0.001; d2 Brood size: χ^2^=7.76, d.f.=1, *P*<0.01; Sex: χ^2^=83.59, d.f.=2, *P*<0.001 Treatment×Age: χ^2^=33.65, d.f.=4, *P*<0.001, Table S1), whereas hatch date had no effect. Post hoc comparisons showed that at 2 days old (i.e. pre-treatment), nestlings of the heat group were slightly lighter than the control group (estimate=0.1±0.03, *P*<0.01) and the cold group (estimate=0.08±0.031, *P*<0.05), while the cold group did not differ from control group (*P*>0.05). At 8 and 14 days old (i.e. post-treatment), no significant differences among treatments were detected (all *P*>0.18). Across ages, larger broods had slightly reduced nestlings body mass (χ^2^=7.76, d.f.=1, *P*<0.01).

### Plasma immunoglobulin concentration

**Fig. 3.**
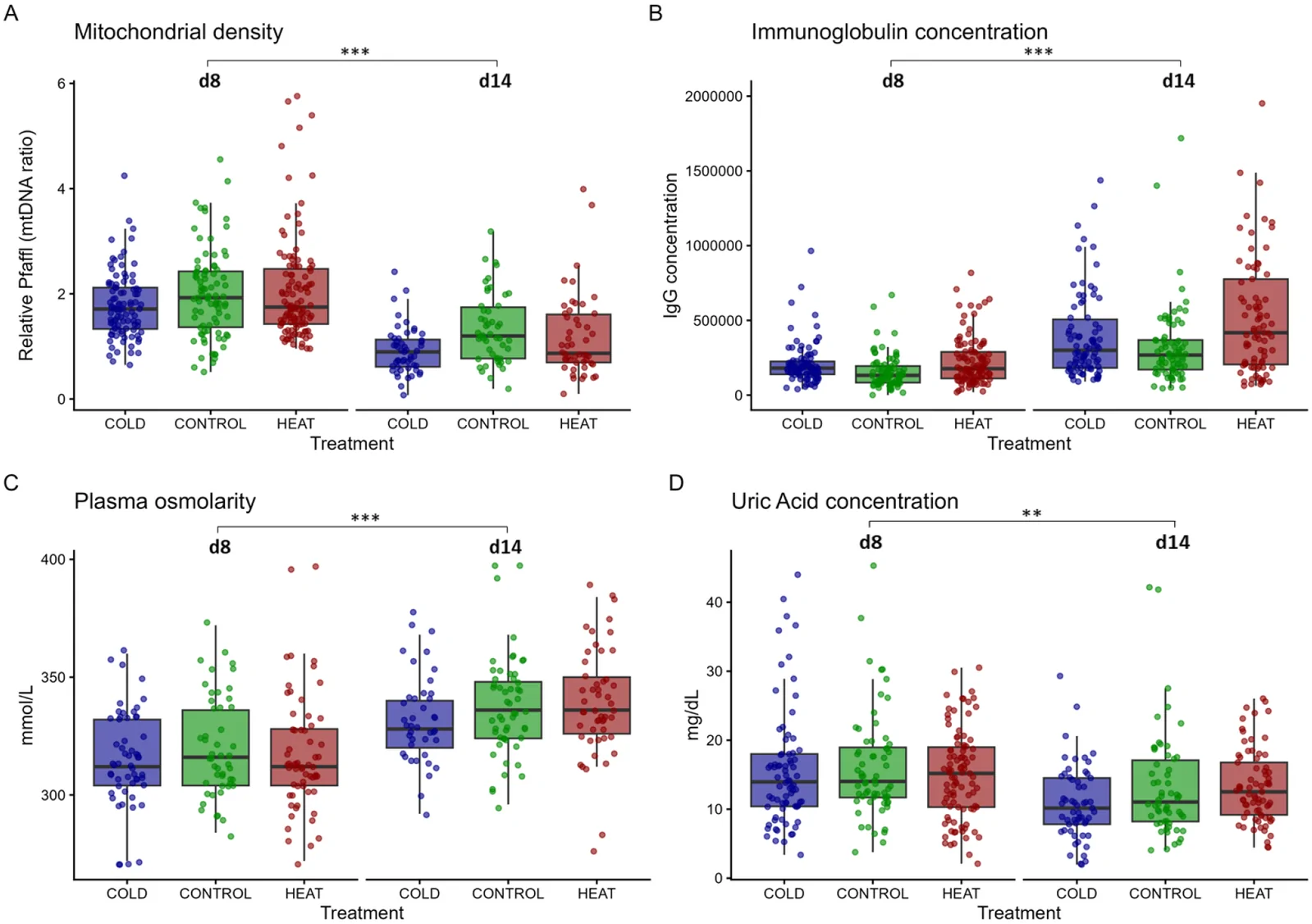
**Effects of thermal treatment on nestling physiological conditions across development.** Boxplots show the distribution of four physiological markers measured at different ages (days post-hatching, 8 or 14) in nestlings from three treatment groups: control (green), cold (blue), and heat (red). (A) Mitochondrial density expressed as relative Pfaffl ratio (mtDNA/nDNA). (B) Immunoglobulin concentration (IgG, arbitrary units). (C) Plasma osmolarity (mmol/l). (D) Uric acid concentration (mg/dl). Boxes represent the interquartile range (IQR), horizontal lines indicate the median, and individual data points are shown as jittered dots.

Within each treatment, IgG levels increased significantly with age, with no difference in rate of increase among treatments (*P*>0.97; Table S2, Fig. 3). However, there was a small but statistically significant overall effect of treatment (χ^2^=6.4, d.f.=2, *P*=0.04). When data from all ages were pooled, pairwise comparisons confirmed overall higher IgG levels in heat compared to controls (Control versus Heat: Estimate±s.e.=−0.38±0.18, *P*=0.04), while the contrast between control and cold was marginally non-significant (Control versus Cold: Estimate±s.e.=−0.34±0.18, *P*=0.06) and no differences appeared between heat and cold groups (Heat versus Cold: Estimate±s.e.=−0.03±0.17, *P*=0.84). Pairwise comparisons within each age class were not significant (all *P*>0.09).

### Uric acid concentration and plasma osmolarity

Uric acid concentration was not significantly affected by treatment or by the treatment×age interaction. However, the mixed-effects model revealed an age effect (χ^2^=7.31, d.f.=1, *P*<0.01) with 14 days old chicks showing lower uric acid concentrations than 8 days old chicks (Estimate±s.e.=−0.23±0.088, *P*<0.01). No significant associations were detected with brood size or mass at day 2 (all *P*>0.3).

Plasma osmolarity was not significantly affected by treatment or by the treatment×age interaction. However, the mixed-effects model revealed a strong effect of age (χ^2^=23.56, d.f.=1, *P*<0.001) with 14 days old chicks showing higher osmolarity than 8 days old chicks (Estimate±s.e.=0.054±0.014, *P*<0.001). Plasma osmolarity increased with hatch date (χ^2^=8.37, d.f.=1, *P*<0.01). No significant associations were detected with brood size or mass at day 2 (all *P*>0.2).

### Mitochondrial density

Relative mitochondria density declined significantly with age (χ^2^=32.68, d.f.=1, *P*<0.001) in all treatments, whereas treatment and its interactions with age were not significant (*P*>0.05), indicating consistent patterns across experimental groups (Table S2, Fig. 3).

#### Parental behavior

Treatment did not affect feeding rate at day 4 (χ^2^=0.39, d.f.=2, *P*=0.68). Neither brood size (χ^2^=2.40, d.f.=1, *P*=0.14), area (χ^2^=0.71, d.f.=9, *P*=0.69), nor time of the day (χ^2^=1.33, d.f.=1, *P*=0.26) had a significant effect on feeding rate.

## DISCUSSION

In this study, our results suggest that modest, environmentally realistic alterations of nest temperature during the early postnatal period in great tits had no effects on nestling survival, morphology, or physiological parameters. These observations may suggest an early-life thermal tolerance to moderate temperature fluctuations in this population, indicating a degree of resilience or plasticity under the tested conditions. Alternatively, these results could also suggest that the temperature manipulation was insufficient to produce measurable effects on the selected variables or that the effects of such manipulation only emerge at a later stage in the life of the organism.

### Nest temperature, experimental manipulation, and parental care

Our experimental manipulation successfully increased nest temperatures by ∼2.3°C in heated boxes between days 2 to 8, whereas chilled boxes were ∼1.3°C cooler than controls (with a slightly stronger short-term cooling effect of ca 2°C). As shown in previous work, modifying nest microclimate in natural conditions is feasible, but often constrained by environmental variability (Andreasson et al., 2018). The pronounced daily and seasonal temperature fluctuations we recorded underscore how dynamic the thermal environment of free-living nestlings is, with natural temperature variations frequently exceeding the 1-2°C differences created here experimentally (e.g. average temperature of the control nestboxes range 13.9-23.1°C, Fig. S1). Such high background variability likely reduced the effective contrast between treatments and may have masked subtle effects. After accounting for temporal variation, heated nests remained significantly warmer and cooled nests significantly cooler than control nests, with treatment differences approaching 2°C during the warmest part of the day. Thus, nestlings experienced distinct thermal environments despite the high variability characteristic of natural nest conditions. Consistent with this interpretation, analyses based on realized nest temperatures rather than treatment identity showed qualitatively similar results (Table S3).

Parental care did not appear to differ between treatments, although brooding duration could not be quantified. We therefore cannot exclude the possibility that parents partially compensated for altered nest temperatures (Rodríguez and Barba, 2016a), which could have further buffered potential effects on nestlings.

### Nestling survival and morphometric parameters

Contrary to our predictions, modest early-life thermal manipulation did not significantly affect nestling survival or morphometric traits. Nestling body mass and wing length varied as expected with age and brood size, but showed no response to the experimental treatments. These findings are consistent with several previous studies reporting no effect of temperature on fledging success or growth (Nord and Nilsson, 2016; Rodriguez and Barba, 2016a,b; Stier et al., 2021; Hsu et al., 2020; Ton et al., 2021; Furic et al., 2026), although others have found reduced body mass under thermal challenges (Rodriguez and Barba, 2016a,b; Corregidor-Castro and Jones, 2021; Woodruff et al., 2025; Satarkar et al., 2026; van de Ven et al., 2020). Notably, these latter studies generally involved longer or stronger temperature differences (4-5°C), approximately twice the magnitude of our manipulation, suggesting that effects on growth may only emerge beyond a certain thermal threshold.

Moreover, variation in mortality was primarily explained by differences among nests, indicating that factors such as microhabitat, parental quality, or predation may play a stronger role than the relatively modest thermal variation imposed here. This interpretation is consistent with experimental studies showing that stressful factors affecting females, including perceived predation risk and socially mediated signals, can have substantial carry-over effects on nestling growth, physiology, and survival (Coslovsky and Richner, 2011; McNew et al., 2024). Additionally, perceived predator exposure during the nestling period can strongly reduce fledging success (Basso and Richner, 2015; Taff et al., 2023), although effects may vary with ecological context and timing (e.g. lack of effects in pied flycatchers, Morosinotto et al., 2019).

Differences among studies may also reflect the timing and duration of thermal exposure. While most experimental periods overlap with early postnatal development, some extend into later stages (e.g. up to day 13-15), when nestlings face higher energetic demands but also develop increasing thermoregulatory capacity (Dawson and O'Connor, 1996). Although early developmental stages are often considered particularly sensitive to environmental conditions, our results support the idea that, under mild thermal challenge, passerine nestlings exhibit sufficient plasticity to adjust growth trajectories in response to environmental conditions (Metcalfe and Monaghan, 2001; Nord and Nilsson, 2016). Moreover, the effects of temperature on growth may be mediated indirectly through ecological factors such as food availability rather than direct thermal constraints. For instance, Satarkar et al. (2026) showed that reduced fledging mass was associated with extreme cold events during the first week of development but also with the combined effects of extreme heat and heavy rainfall, whereas heat alone had no significant impact. Warmer conditions can increase insect activity and visibility, potentially enhancing prey availability for provisioning parents (Schöll et al., 2016), while sufficiently warm nest conditions may reduce brooding time and allow parents to allocate more effort to foraging (Dawson et al., 2005; Rodríguez and Barba, 2016b). Overall, this highlights the context-dependent nature of thermal effects, with potentially neutral or even beneficial outcomes under specific environmental conditions but detrimental when combined with other climatic conditions such as precipitation (Rodríguez and Barba, 2016b).

### Physiological responses

We found no significant treatment effects on plasma osmolarity, uric acid concentration, mitochondrial density or immunoglobulin levels, although all traits changed with age as expected, reflecting variation in the physiological development of nestlings. The lack of short-term changes in these physiological markers, which are often more sensitive to environmental changes than growth or survival, further suggests that nestlings maintained physiological homeostasis under the imposed thermal variation, possibly aided by parental buffering.

Previous experimental studies have shown that elevated nest temperatures can affect physiological pathways, including metabolism, mitochondrial function, stress physiology, and hematological parameters (Ardia, 2013; Ton et al., 2021; Stier et al., 2021; Furic et al., 2026). These responses are generally interpreted as reflecting increased thermoregulatory and metabolic demands under elevated temperatures, which can in turn influence energy allocation and physiological conditions. However, the magnitude and direction of these effects appear to depend on the intensity and duration of thermal exposure, with stronger or prolonged thermal challenges more consistently inducing detectable effects (Nord and Nilsson, 2016; Andreasson et al., 2018).

All traits changed with age as expected, reflecting natural variation in the physiological development of nestlings. Plasma osmolarity increased with age, likely reflecting maturation of osmoregulatory function and shifts toward a more protein-rich, less water-rich diet (Andrewartha et al., 2011; Gibb and Betts, 1963; Naef-Daenzer, 2000). In contrast, uric acid levels declined between day 8 and day 14, suggesting improved metabolic efficiency as nestlings develop endothermy (Dawson and O'Connor, 1996; Cohen et al., 2008; Nord and Nilsson, 2016). Mitochondrial density also decreased over time, consistent with reduced cellular metabolic demand as growth rates slow and tissues mature (Cossin-Sevrin et al., 2022, 2023; Stier et al., 2021; Salmón et al., 2026). Finally, increasing immunoglobulin levels reflect the progressive development of the immune system, as nestlings transition from maternally derived to endogenous antibodies (Grindstaff et al., 2003; King et al., 2010). Taken together, these patterns indicate that our thermal manipulation remained within a physiologically tolerable range and did not disrupt normal hydration, metabolic or immune development (West-Eberhard, 2003).

### Implications for early-life thermal challenge and climate change

Our study suggests that moderate deviations in nest temperature such as those observed through microclimate variation or small-scale heatwaves or cold snaps may have limited short-term impacts on nestling growth and physiology. Our results contribute to a growing body of work on the developmental effects of thermal environments in wild birds (Rodriguez and Barba, 2016a,b; Andreasson et al., 2018; Taff et al., 2026; Taff and Shipley, 2023; Satarkar et al., 2026). Experimental studies in other species (e.g. pied flycatchers, ±2°C, Furic et al., 2026; blue tits, +1.5 to +3°C, Rodríguez and Barba, 2016a,b) have reported both immediate and latent effects of early-life thermal conditions, even following relatively small temperature manipulations. Great tits are a widespread generalist species occupying a broad range of climatic conditions, and previous work has shown that thermal reaction norms can vary among populations (Broggi et al., 2005), suggesting that developmental responses may be shaped by local environmental history. Within this context, the absence of short-term effects observed here may reflect population-specific sensitivity to ecologically realistic thermal variation. Additionally, this study was conducted within a single great tit population, and thus captures within-population responses to thermal variation rather than differences in reaction norms among populations. Although population-specific environmental history may shape developmental plasticity in this species, testing such hypotheses requires comparative approaches across multiple populations. However, larger or more prolonged extreme events could exceed the buffering capacity of both nestlings and parents, potentially leading to stronger fitness consequences (Lindström, 1999; Salaberria et al., 2014; Rodriguez and Barba, 2016a,b; Woodruff et al., 2025; Moreno et al., 2015; Levillain et al., 2025).

Because thermogenic capacity develops gradually during the first week after hatching and homeothermy is not yet fully established, thermal conditions experienced within the nest during this period may influence nestling physiology even in the absence of temperatures that would be considered stressful for adult birds (Engert et al., 2025). Future studies combining similar temperature manipulations with direct measurements of body temperature and metabolic rate could provide valuable insight into the physiological mechanisms underlying nestling responses to thermal variation.

A key concept linking early environmental conditions to later-life outcomes is developmental plasticity i.e. the ability of an organism to adjust its development in response to environmental cues (West-Eberhard, 2003). Plastic responses to temperature variations may allow nestlings to buffer immediate thermal challenges by adjusting thermoregulatory capacity, metabolic pathways or immune function (Metcalfe and Monaghan, 2001; Nord and Nilsson, 2016). However, such adjustments can also involve trade-offs that carry delayed costs, for example on growth or future reproduction (Metcalfe and Monaghan, 2001; Andreasson et al., 2018). Even if short-term growth, survival, and some physiological markers seem unaffected, early-life thermal challenge could affect other molecular or endocrine pathways with delayed consequences. Moderate temperature modifications have been shown to affect heat shock protein gene expression (Xie et al., 2018), corticosterone levels (Newberry and Swanson, 2018), or telomere length (Eastwood et al., 2023; Stier et al., 2021; Furic et al., 2026), a biomarker of somatic state and ageing. Other recently suggested mechanisms for mediating long-lasting effects of early (thermal) conditions also include epigenetic mechanisms and the gut microbiome (e.g. Ruuskanen, 2024). Additionally, incorporating genomic tools (Gerson et al., 2024) could further advance our understanding of the mechanistic pathways through which thermal challenge influences development.

In our study, no detectable effects were observed either at the end of the treatment (d8) or 6 days later (d14). Nevertheless, it is important to consider that the impact of early-life challenges can remain latent and only manifest when individuals are later exposed to additional environmental or physiological challenges (Wada and Coutts, 2021). Future work should therefore examine long-term fitness outcomes under ecologically relevant conditions. Understanding how early-life thermal events shape fitness requires examining more extreme or prolonged temperature challenges, as well as tracking individuals into adulthood to assess long-term performance, reproductive success, and survival. Such integrative approaches will be essential for identifying the mechanisms that determine species' resilience or vulnerability to increasingly variable climates.

## Supplementary Material



10.1242/biolopen.062670_sup1 Supplementary information
