# Supplementary material for: Impacts of early-life thermal challenge on development, survival, and physiology in wild great tits
Source: Biol Open. 2026 Sep 11;15(9):bio062670. doi: 10.1242/bio.062670 (PMC13602370; doi:10.1242/bio.062670)
Supplement: Supplementary file 1 [file biolopen-15-062670-s1.pdf]

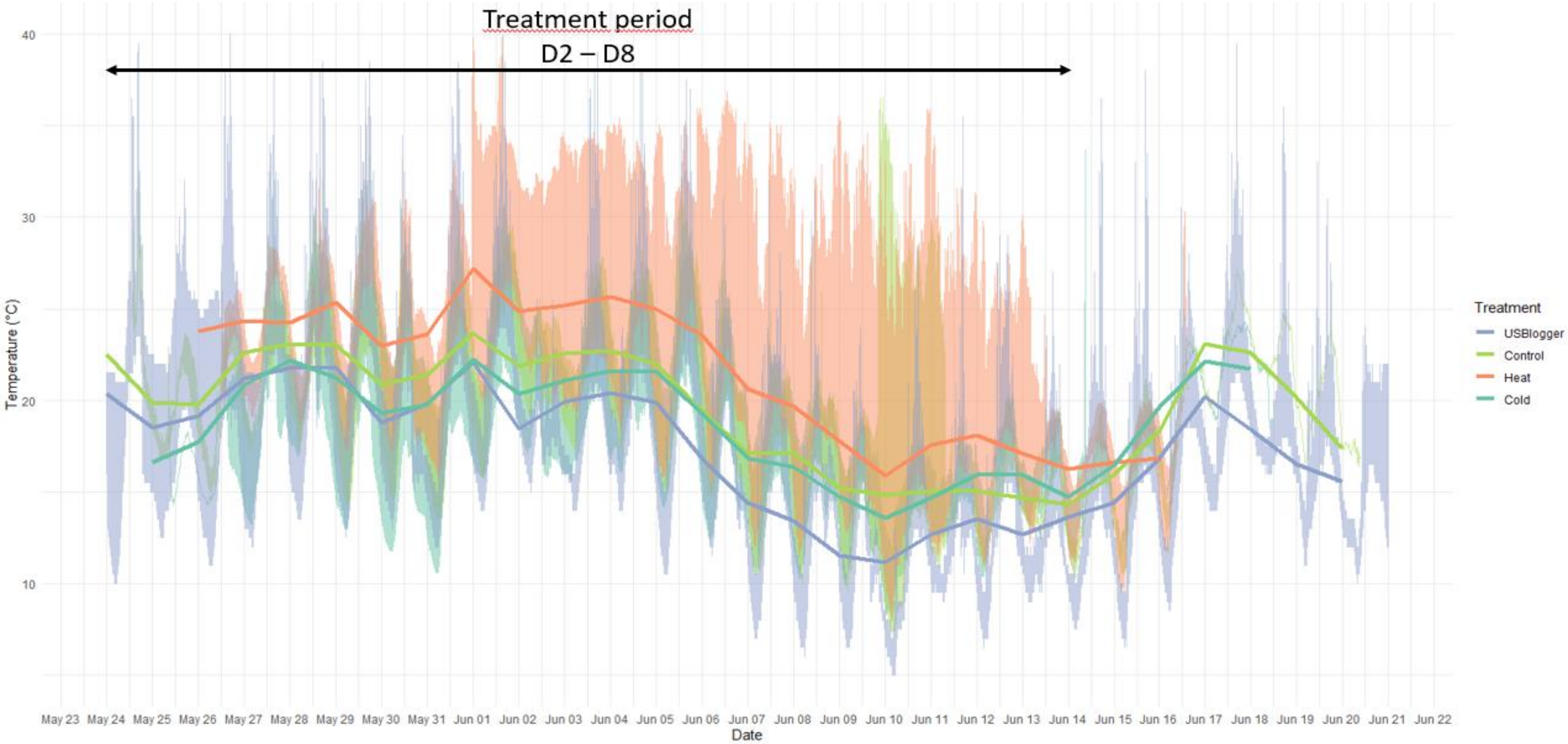

Fig. S1. Daily temperature variation per treatment between day 2 and day 14 for all nest boxes, and average daily ambient temperature variation for all sites (USBlogger).

Table S1. Summary of linear mixed-effects models for nestlings' mass and wing length. Fixed effects are shown with χ^2 values (Type III Wald tests) or estimate, degrees of freedom, and p-values. Post-hoc treatment contrasts are included for mass where the Treatment × Age interaction was significant.

Trait	Fixed Effect	χ^2	Estimate ± SE	df	p-value	Post-hoc / Notes
**Mass (log)**	Treatment	11,42	-	2	**<0.01**	See contrasts below
	Age	4392.4	-	2	**<0.001**	Mass increases with age
	Sex	83.59	-	2	**<0.001**	Males heavier than females or unknown sex
	Hatch day	3.93	-	1	0.05	NS
	d2 Brood size	7.76	-	1	**<0.01**	Negative effect on mass
	Hour of capture	21.72	-	1	**<0.001**	Positive effect on mass
	Treatment × Age	33.65	-	4	**<0.001**	Significant interaction
**Post-hoc (emmeans)**	d2: Control vs Heat	-	0.1 ± 0.03	-	**<0.01**	Heat nestlings lighter than Control
	d2: Cold vs Heat	-	0.08 ± 0.03	-	**<0.05**	Heat nestlings lighter than Cold
	d8: all contrasts	-	-	-	>0.5	NS
	d14: all contrasts	-	-	-	>0.6	NS
**Wing length (log)**	Treatment	2.41	-	2	0.3	NS
	Age	1575	-	1	**<0.001**	Wing lenght increase with age
	Sex	25.69	-	2	**<0.001**	Males have longer wings than females or unknown sex
	Hatch day	0.32	-	1	0.57	NS
	d2 Brood size	0.003	-	1	0.96	NS
	Hour of capture	0.81	-	1	0.37	NS
	Treatment × Age	1.09	-	2	0.58	NS
**Wing: Post-hoc (emmeans)**	All age-treatment contrasts	-	-	-	>0.2	NS

Biology Open • Supplementary information

Table S2. Summary of linear mixed model results (Type III Wald χ^2 tests) for physiological response variables measured in chicks at days 8 and 14 under three thermal treatments. Post-hoc treatment contrasts are included for Plasma immunoglobulin concentration where the Treatment was significant.

Response variable	Fixed effect	χ^2	Estimate ± SE	df	p-value	Direction / post-hoc summary
**Mitochondrial density**	Treatment	2.08	-	2	0.35	NS
	Age (d14 vs d8)	32.68	-	1	**<0.001**	Lower at day 14
	Hatch day	0.26	-	1	0.61	NS
	Brood d2	0.15	-	1	0.7	NS
	Treatment × Age	2.08	-	2	0.35	NS
**Plasma immunoglobulin concentration**	Treatment	6.4	-	2	**0.04**	See contrasts below
	Age (d14 vs d8)	61.85	-	1	**<0.001**	Higher at day 14
	Hatch day	3.26	-	1	0.07	NS
	Brood d2	0.01	-	1	0.99	NS
	Treatment × Age	0.06	-	2	0.972	NS
**Post-hoc (emmeans)**	Control vs. Heat	-	−0.38 ± 0.18	-	**0.04**	Higher in Heat than Control
	Control vs. Cold	-	−0.34 ± 0.18	-	0.06	Marginal trend
**Uric acid**	Treatment	0.89	-	2	0.64	NS
	Age (d14 vs d8)	**7.31**	-	1	**<0.01**	Lower at day 14
	Hatch day	2.04	-	1	0.15	NS
	Brood d2	3.5	-	1	0.06	Marginal trend
	Treatment × Age	5.38	-	2	0.07	NS
	Plasma hemolyse	0.73	-	1	0.39	NS
**Plasma osmolarity**	Treatment	1.11	-	2	0.57	NS
	Age (d14 vs d8)	23.56	-	1	**<0.001**	Higher at day 14
	Hatch day	8.37	-	1	**<0.01**	Increase with Hatch date
	Brood d2	2.52	-	1	0.11	NS
	Treatment × Age	0.29	-	2	0.87	NS

Table S3. Pairwise comparisons of nest temperature treatment (CONTROL, COLD, HEAT) on nestling body mass (log-transformed, wing length, mitochondrial density, immunoglobulin concentration, plasma osmolarity and uric acid within each age class (D2, D8, D14) and hatching schedule (Early, Late). Estimates represent differences between treatment levels (least-squares means) from the linear mixed-effects model, with Tukey-adjusted p-values.

Trait	Age	Schedule	contrast	Estimate	SE	df	Statistic	**p_value**
Body mass (log)	2	Early	CONTROL - COLD	0.022	0.047	57.1	0.46	**0.888**
	2	Early	CONTROL - HEAT	0.118	0.047	58.9	2.49	**0.041**
	2	Early	COLD - HEAT	0.096	0.045	57.3	2.12	**0.095**
	8	Early	CONTROL - COLD	-0.044	0.049	71.5	-0.88	**0.653**
	8	Early	CONTROL - HEAT	0.001	0.05	72.9	0.01	**1.000**
	8	Early	COLD - HEAT	0.044	0.047	67.2	0.94	**0.620**
	14	Early	CONTROL - COLD	-0.038	0.05	74.4	-0.77	**0.724**
	14	Early	CONTROL - HEAT	-0.018	0.051	78.3	-0.36	**0.932**
	14	Early	COLD - HEAT	0.02	0.049	74	0.41	**0.911**
	2	Late	CONTROL - COLD	0.022	0.047	57.1	0.46	**0.888**
	2	Late	CONTROL - HEAT	0.118	0.047	58.9	2.49	**0.041**
	2	Late	COLD - HEAT	0.096	0.045	57.3	2.12	**0.095**
	8	Late	CONTROL - COLD	-0.044	0.049	71.5	-0.88	**0.653**
	8	Late	CONTROL - HEAT	0.001	0.05	72.9	0.01	**1.000**
	8	Late	COLD - HEAT	0.044	0.047	67.2	0.94	**0.620**
	14	Late	CONTROL - COLD	-0.038	0.05	74.4	-0.77	**0.724**
	14	Late	CONTROL - HEAT	-0.018	0.051	78.3	-0.36	**0.932**
	14	Late	COLD - HEAT	0.02	0.049	74	0.41	**0.911**
Wing length (log)	8	Early	CONTROL - COLD	0.054	0.042	39.5	1.29	**0.410**
	8	Early	CONTROL - HEAT	0.07	0.043	39.8	1.64	**0.242**
	8	Early	COLD - HEAT	0.016	0.04	38	0.4	**0.916**
	14	Early	CONTROL - COLD	0.052	0.042	40.7	1.22	**0.446**
	14	Early	CONTROL - HEAT	0.051	0.043	41.7	1.18	**0.470**
	14	Early	COLD - HEAT	-0.001	0.041	40.7	-0.02	**1.000**
	8	Late	CONTROL - COLD	0.054	0.042	39.5	1.29	**0.410**
	8	Late	CONTROL - HEAT	0.07	0.043	39.8	1.64	**0.242**
	8	Late	COLD - HEAT	0.016	0.04	38	0.4	**0.916**
	14	Late	CONTROL - COLD	0.052	0.042	40.7	1.22	**0.446**
	14	Late	CONTROL - HEAT	0.051	0.043	41.7	1.18	**0.470**
	14	Late	COLD - HEAT	-0.001	0.041	40.7	-0.02	**1.000**
Mitochondrial density	8	Early	CONTROL - COLD	0.224	0.215	37.9	1.04	**0.557**
	8	Early	CONTROL - HEAT	-0.009	0.219	38	-0.04	**0.999**
	8	Early	COLD - HEAT	-0.233	0.204	36.7	-1.14	**0.498**
	14	Early	CONTROL - COLD	0.433	0.229	48.8	1.89	**0.154**
	14	Early	CONTROL - HEAT	0.131	0.235	49.7	0.56	**0.843**
	14	Early	COLD - HEAT	-0.301	0.224	51.6	-1.35	**0.376**
	8	Late	CONTROL - COLD	0.224	0.215	37.9	1.04	**0.557**
	8	Late	CONTROL - HEAT	-0.009	0.219	38	-0.04	**0.999**
	8	Late	COLD - HEAT	-0.233	0.204	36.7	-1.14	**0.498**
	14	Late	CONTROL - COLD	0.433	0.229	48.8	1.89	**0.154**
	14	Late	CONTROL - HEAT	0.131	0.235	49.7	0.56	**0.843**
	14	Late	COLD - HEAT	-0.301	0.224	51.6	-1.35	**0.376**

Biology Open • Supplementary information

IgG concentration (log)	8	Early	CONTROL - COLD	-0.41	0.199	44.3	-2.06	**0.109**
	8	Early	CONTROL - HEAT	-0.361	0.202	44	-1.79	**0.185**
	8	Early	COLD - HEAT	0.049	0.19	42.8	0.26	**0.964**
	14	Early	CONTROL - COLD	-0.28	0.202	46.8	-1.39	**0.356**
	14	Early	CONTROL - HEAT	-0.387	0.207	48.1	-1.88	**0.157**
	14	Early	COLD - HEAT	-0.107	0.197	48.4	-0.55	**0.849**
	8	Late	CONTROL - COLD	-0.41	0.199	44.3	-2.06	**0.109**
	8	Late	CONTROL - HEAT	-0.361	0.202	44	-1.79	**0.185**
	8	Late	COLD - HEAT	0.049	0.19	42.8	0.26	**0.964**
	14	Late	CONTROL - COLD	-0.28	0.202	46.8	-1.39	**0.356**
	14	Late	CONTROL - HEAT	-0.387	0.207	48.1	-1.88	**0.157**
	14	Late	COLD - HEAT	-0.107	0.197	48.4	-0.55	**0.849**
Plasma osmolarity (log)	8	Early	CONTROL - COLD	0.023	0.026	71	0.91	**0.636**
	8	Early	CONTROL - HEAT	-0.012	0.026	66.3	-0.49	**0.878**
	8	Early	COLD - HEAT	-0.036	0.024	65.4	-1.47	**0.310**
	14	Early	CONTROL - COLD	0.005	0.026	83.4	0.19	**0.980**
	14	Early	CONTROL - HEAT	0.006	0.026	73.1	0.24	**0.970**
	14	Early	COLD - HEAT	0.001	0.026	92.7	0.04	**0.999**
	8	Late	CONTROL - COLD	0.023	0.026	71	0.91	**0.636**
	8	Late	CONTROL - HEAT	-0.012	0.026	66.3	-0.49	**0.878**
	8	Late	COLD - HEAT	-0.036	0.024	65.4	-1.47	**0.310**
	14	Late	CONTROL - COLD	0.005	0.026	83.4	0.19	**0.980**
	14	Late	CONTROL - HEAT	0.006	0.026	73.1	0.24	**0.970**
	14	Late	COLD - HEAT	0.001	0.026	92.7	0.04	**0.999**
Uric acid (log)	8	Early	CONTROL - COLD	0.049	0.107	62.3	0.46	**0.891**
	8	Early	CONTROL - HEAT	0.087	0.107	58.3	0.81	**0.696**
	8	Early	COLD - HEAT	0.038	0.1	55.4	0.38	**0.922**
	14	Early	CONTROL - COLD	0.156	0.115	77.9	1.36	**0.365**
	14	Early	CONTROL - HEAT	-0.042	0.115	73	-0.37	**0.928**
	14	Early	COLD - HEAT	-0.199	0.111	80.7	-1.79	**0.181**
	8	Late	CONTROL - COLD	0.049	0.107	62.3	0.46	**0.891**
	8	Late	CONTROL - HEAT	0.087	0.107	58.3	0.81	**0.696**
	8	Late	COLD - HEAT	0.038	0.1	55.4	0.38	**0.922**
	14	Late	CONTROL - COLD	0.156	0.115	77.9	1.36	**0.365**
	14	Late	CONTROL - HEAT	-0.042	0.115	73	-0.37	**0.928**
	14	Late	COLD - HEAT	-0.199	0.111	80.7	-1.79	**0.181**

Biology Open • Supplementary information

Table S4. Effects of nest temperature on nestlings' body mass, wing length, mitochondrial density, immunoglobulin concentration, plasma osmolarity and uric acid. "Slope vs. zero" rows test whether traits respond to nest temperature within each age class; p-values for slope differences are Tukey-adjusted for 3 comparisons.

Trait	Age	Slope	SE	df	CI_95	Statistic	**p_value**
Body mass (log)	2	0.035	0.022	48.1	[-0.079, 0.009]	-1.62	**0.112**
	8	0.009	0.024	62.3	[-0.038, 0.057]	0.4	**0.692**
	14	0.02	0.024	63.8	[-0.027, 0.067]	0.84	**0.401**
Wing length (log)	8	0.014	0.024	37.4	[-0.034, 0.063]	0.6	**0.553**
	14	0.015	0.024	38.2	[-0.033, 0.064]	0.64	**0.524**
Mitochondrial density	8	0.013	0.109	38.8	[-0.234, 0.207]	-0.12	**0.904**
	14	0.015	0.113	47	[-0.212, 0.242]	0.13	**0.896**
IgG concentration (log)	8	0.167	0.119	37.9	[-0.074, 0.409]	1.41	**0.168**
	14	0.169	0.116	41.7	[-0.065, 0.403]	1.46	**0.153**
Plasma osmolarity (log)	8	0.014	0.014	57.4	[-0.013, 0.042]	1.06	**0.293**
	14	0.004	0.014	56.1	[-0.031, 0.023]	-0.3	**0.766**
Uric acid (log)	8	0.436	0.878	44.6	[-1.334, 2.205]	0.5	**0.622**
	14	1.639	0.879	50	[-3.404, 0.127]	-1.86	**0.068**

Biology Open • Supplementary information
