# Supplementary material for: Impacts of early-life thermal challenge on development, survival, and physiology in wild great tits
Source: Biol Open. 2026 Sep 11;15(9):bio062670. doi: 10.1242/bio.062670 (PMC13602370; doi:10.1242/bio.062670)
Supplement: Supplementary file 2 [file biolopen-15-062670_review_history.pdf]

Impacts of early-life thermal challenge on development, survival, and physiology in wild great tits

Clemence Furic, Charli S. Davies, Jenna Palttala, Valentina Giongo, Loan Hognon-Pelletier, Suvi Ruuskanen, Sophie Reichert and Julie Fleitz
10.1242/bio.062670

Editor: Kendra J. Greenlee

Review timeline

Original submission:	30 April 2026
Editorial decision:	5 May 2026
First revision received:	15 July 2026
Accepted:	16 July 2026

Original submission

First decision letter

MS ID#: bio.062670

MS Title: Impacts of early-life thermal stress on development, survival, and physiology in wild great tits

Authors: Julie Fleitz; Clemence Furic; Charli S. Davies; Jenna Palttala; Valentina Giongo; Loan Hognon-Pelletier; Suvi Ruuskanen; Sophie Reichert

I have now reached a decision on the above manuscript.

The reviewer reports are shown at the bottom of this email.

As you will see, the reviewers raised a number of substantial criticisms that prevent me from accepting the paper at this stage. I checked whether their comments were valid. The experimental design was acceptable, but I do agree with both reviewers that the interpretation of the warm treatment as a stressful treatment is misleading without further evidence to show that +2C is ecologically important compared to natural variations that they are normally exposed to (Fig. 1 and Fig. S1). The logical flow and structure could be clearer, as reviewer 2 pointed out.

I will offer the opportunity for the authors to either clarify and justify the interpretations of the study, or tone down findings and to include limitations. It was not clear why/+ 2C average was chosen and how it compares against background variation, as there is a lot of overlap with the control treatment (especially the cold treatment). If you think that you can deal satisfactorily with the criticisms on revision, I would be pleased to see a revised manuscript. We would then return it to the reviewers.

At this stage, we also ask you to ensure your manuscript complies with our formatting guidelines.

Please upload both a 'clean' version of your Word file, along with a highlighted version clearly showing where you have made changes in the revised manuscript. Please avoid using 'Track changes' in Word files as these are lost in PDF conversion.

© 2026. Published by The Company of Biologists under the terms of the Creative Commons Attribution License (https://creativecommons.org/licenses/by/4.0/).

I should be grateful if you would also provide a point-by-point response detailing how you have dealt with the points raised by the reviewers in the 'Response to Reviewers' box. Please attend to all of the reviewers' comments. If you do not agree with any of their criticisms or suggestions please explain clearly why this is so.

Reviewer 1

Comments for the author

The authors investigated the effects of experimentally increased and decreased nest temperatures on multiple traits measured in nestlings. To this end, they used heating and cooling pads placed inside nests. Overall, the results provide no evidence for an effect of temperature manipulation on nestling traits.

The topic is timely and relevant, particularly in the context of ongoing global temperature changes. Moreover, the combination of an experimental approach conducted in the wild with the measurement of multiple endpoints represents a clear strength of the study. However, I identify one major limitation that may be difficult to address.

The authors interpret their treatments as inducing heat stress (in warmed nests) and cold stress (in cooled nests). This assumption is not sufficiently justified, which complicates the interpretation of the results. In particular, information on the thermoneutral zone of the study species (great tit) would be essential to assess whether the applied temperature manipulations are likely to elicit physiological stress. In addition, data on how body temperature and metabolic rate vary across ambient temperatures would help determine the thresholds at which thermal conditions become stressful for nestlings.

In the absence of such information, it remains unclear whether the experimental treatments actually imposed thermal stress. It is possible that the manipulated temperatures remained within the thermoneutral zone, which could explain the lack of detectable effects. If this is the case, the experimental design may not be suitable for testing the stated hypotheses.

further comments:

given my major concern, I am not going to provide more specific comments at this stage. However, I think that the temperature regimes within experimental groups would need to be compared. For figure 1, it seems that there is some overlap in temperature between nests of different experimental groups.

Reviewer 2

Comments for the author

Note on raw data: I did not see raw data appended in the reviewer portal.

General comments:

In this study, the authors manipulated nest temperature in wild nesting Great Tits to study whether and how heat and cold stress affect development and physiology.

Despite honesty about how modest temperature manipulations were, I would have liked to see the authors put this into more context and better define why these small-scale changes were "ecologically relevant". Based on what the authors mentioned about this Great Tit population being a high-latitude population accustomed to large temperature fluctuations, the temperature treatments induced (target: +/- 2 degrees C) perhaps too subtle to produce the signal the authors had anticipated.

© 2026. Published by The Company of Biologists under the terms of the Creative Commons Attribution License (https://creativecommons.org/licenses/by/4.0/).

Various parts of the introduction and discussion were somewhat superficial and would be strengthened by inclusion of relevant work from other wild bird systems. For example, research teams working on Tree Swallows have actively manipulated temperatures in nest boxes for years and have assessed physiological (mostly stress parameters) and developmental impacts on nestlings (e.g., Taff et al. 2026, Current Biology; see other work by Taff and Vitousek). Additionally, several recent studies have looked at longer term impacts of inclement temperature fluctuations on fitness (Taff and Shipley 2023, Nature Communications https://www.nature.com/articles/s41467-023-43071-y), as well asa phenology, morphology, and aspects of reproduction (e.g., Stager et al. 2026, Nature Ecology and Evolution). Finally, theoretical models about resilience and robustness (e.g. Vitousek et al. 2025, Trends in Ecology and Evolution) would provide a conceptual basis for the current work that would better ground the authors' predictions.

It would additionally be helpful for the authors to discuss how genetic background could impact scope of response and capacity for developmental plasticity within nestlings.

Specific comments:

Lines 57-59: But see work by Tree Swallow folks, for example. Also see Xue et al. 2025, Nature Communications.

Line 72: the phrase "thermoregulatory capacity" should be better defined - what is considered within the 'normal' scope for adult birds, and how does this vary with genetic background and environment?

Lines 73-74: The authors should provide more information on how these rough temperature thresholds were selected. Presumably, 2 degrees C is within the normal range of variation that a nestling might experience when a parent leaves the nest to forage, no? The phrase 'ecologically relevant' therefore seems context dependent. Furthermore, what is relevant to a cavity nester may not be ecologically relevant to an open cup nester in a less protected environment. In short, this small-scale temperature manipulation seems so slight that it would be unlikely to impact nestlings, so some justification for why it was chosen is warranted.

Lines 86-87: Why did the authors predict that heat- and cold-stressed birds would both suffer reduced body mass?

Lines 104-107: This sentence seems to provide further evidence that increases or decreases in temperature of 2 degrees C may have negligible impacts on nestlings.

Lines 108-109: Do females incubate continuously or do they leave to forage?

Lines 121-122: Abstract lists temperature reduction as approximately -1.3 degrees C; 2 degrees C is listed here.

Lines 124-125: specify the precision of the ibuttons used.

Lines 224-233: It would be useful to describe how temperatures inside each nest box varied relative to ambient external temperature (seasonality is a covariate here). Did the authors examine growth and development in relation to the timing of nesting during the season, e.g., early vs late nesters?

Line 305: Add the word "modest" before "early life thermal manipulation".

Lines 315-317: Several relevant papers to cite here on the importance of parent quality and social signal on nestling development (e.g., McNew et al. 2024 JEB, Taff et al. 2023 Journal of Experimental Zoology Part A: Ecological and Integrative Physiology - both papers about manipulation of social signal and downstream responses), as well as the effect of perceived predation on nestling development (e.g., McNew et al. 2024, Ecology https://drive.google.com/file/d/1OeOqqtrj_WaWi4GeVHgV9zgPxF1BGGdM/view)

Reviewer's Responses to Questions

© 2026. Published by The Company of Biologists under the terms of the Creative Commons Attribution License (https://creativecommons.org/licenses/by/4.0/).

Experimental quality

Are experiments performed using appropriate methods that will answer the question (or test the hypothesis or support the observations) posed by the authors? Is the right tool used for the job?
If 'No', please indicate reasons in Comments for Author box below.
Reviewer #1:

- No

Reviewer #2:

- Yes

Were the data analyzed using appropriate statistical tests?
If 'No', please indicate reasons in Comments for Author box below.
Reviewer #1:

- Yes

Reviewer #2:

- Yes

Reproducibility

Were experiments performed using adequate number of biological replicates?
If 'No', please indicate reasons in Comments for Author box below.
Reviewer #1:

- Yes

Reviewer #2:

- Yes

Is there sufficient raw data to assess the rigor of the analysis?
If 'No', please indicate reasons in Comments for Author box below.
Reviewer #1:

- Yes

Reviewer #2:

- No

Does the methods section provide sufficient detail to permit reproducibility?
If 'No', please indicate reasons in Comments for Author box below.
Reviewer #1:

- Yes

Reviewer #2:

- Yes

Completeness

Are the manuscript's conclusions supported by the data?
If 'No', please indicate reasons in Comments for Author box below.
Reviewer #1:

- No

Reviewer #2:

© 2026. Published by The Company of Biologists under the terms of the Creative Commons Attribution License (https://creativecommons.org/licenses/by/4.0/).

- Yes

Scholarship

Do the authors cite and discuss the merits of data that would argue for and against their conclusion?
If 'No', please indicate reasons in Comments for Author box below.
Reviewer #1:
- Yes

Reviewer #2:
- No

Does the manuscript title & abstract accurately reflect the contents of the manuscript, without hyperbole?
If 'No', please indicate reasons in Comments for Author box below.
Reviewer #1:
- Yes

Reviewer #2:
- Yes

First revision

Author response to reviewers' comments

Reviewer 1: The authors investigated the effects of experimentally increased and decreased nest temperatures on multiple traits measured in nestlings. To this end, they used heating and cooling pads placed inside nests. Overall, the results provide no evidence for an effect of temperature manipulation on nestling traits.

The topic is timely and relevant, particularly in the context of ongoing global temperature changes. Moreover, the combination of an experimental approach conducted in the wild with the measurement of multiple endpoints represents a clear strength of the study. However, I identify one major limitation that may be difficult to address.

The authors interpret their treatments as inducing heat stress (in warmed nests) and cold stress (in cooled nests). This assumption is not sufficiently justified, which complicates the interpretation of the results. In particular, information on the thermoneutral zone of the study species (great tit) would be essential to assess whether the applied temperature manipulations are likely to elicit physiological stress.

Authors: We thank the reviewer for this important point. In this study, our objective was not to expose nestlings to temperatures below / above their thermal limits, but rather to experimentally simulate ecologically realistic temperature deviations (±2°C), consistent with near-term climate projections, and assess their consequences for nestling morphology, survival, and physiology. The absence of detectable effects across multiple traits suggests that these temperature differences remained within the tolerance range of early-stage nestlings in this population, a result that is itself biologically informative.

To explicitly address this point, the following information is now added to the introduction section L. 41-46: "For most passerines, the thermoneutral zone (TNZ) lies approximately between 15 and 35°C (Gavrilov and Dolnik, 1985; Playà-Montmany et al., 2021; Cabello-vergel et al., 2022). However, this concept does not translate directly to early altricial nestlings, which have not yet achieved endothermic competence and are largely dependent on ambient nest environment (i.e.

© 2026. Published by The Company of Biologists under the terms of the Creative Commons Attribution License (https://creativecommons.org/licenses/by/4.0/).

parental brooding and nest microclimate) for thermoregulation (Price & Dzialowski 2018; Katzenberger et al. 2015; Boyles et al. 2011; Dawson and O'Connor 1996)."
Additionally, we acknowledge that the terms "heat stress" and "cold stress" may imply a deviation beyond the TNZ and that the term "thermal stress" may overstate the nature of the treatment. We have replaced it throughout the manuscript with "thermal challenge," which more accurately reflects our experimental design without presupposing a physiological stress response.

Reviewer 1: In addition, data on how body temperature and metabolic rate vary across ambient temperatures would help determine the thresholds at which thermal conditions become stressful for nestlings.

In the absence of such information, it remains unclear whether the experimental treatments actually imposed thermal stress. It is possible that the manipulated temperatures remained within the thermoneutral zone, which could explain the lack of detectable effects. If this is the case, the experimental design may not be suitable for testing the stated hypotheses.

Authors: We agree that direct measurements of nestling body temperature and metabolic rate would strengthen causal inference and we have now acknowledged this limitation L. 409-411. However, the concept of a thermoneutral zone, as defined for resting homeothermic adults, is not directly applicable to great tit nestlings during the first week after hatching. At this stage, altricial nestlings are still developing thermoregulatory capacity and remain strongly influenced by nest microclimate and parental brooding.
Recent work in blue tits showed that nestlings between 4 and 8 days of age are not yet capable of sustained homeothermy (Engert et al., 2025). Consequently, precise thermal-stress thresholds are difficult to define for the age classes studied here. Nevertheless, experimental manipulations in closely related species have demonstrated that variation in nest temperature can influence nestling body temperature and growth, indicating that early developmental stages are physiologically responsive to thermal conditions (Andreasson et al., 2018). Future studies combining temperature manipulations with direct measurements of body temperature and metabolic rate would help clarify the mechanisms underlying these responses.

We have added a paragraph to the Discussion section L. 406-411 to explicitly address this point: "Because thermogenic capacity develops gradually during the first week after hatching and homeothermy is not yet fully established, thermal conditions experienced within the nest during this period may influence nestling physiology even in the absence of temperatures that would be considered stressful for adult birds (Engert et al. 2024). Future studies combining similar temperature manipulations with direct measurements of body temperature and metabolic rate could provide valuable insight into the physiological mechanisms underlying nestling responses to thermal variation."

Reviewer 1: further comments: given my major concern, I am not going to provide more specific comments at this stage. However, I think that the temperature regimes within experimental groups would need to be compared. For figure 1, it seems that there is some overlap in temperature between nests of different experimental groups.

Authors: The overlap in raw temperature distributions visible in Figure 1 reflects the high natural thermal variability inherent to free-living nest environments, where daily and seasonal fluctuations of 9°C or more were recorded within the control group alone. This background variability inevitably produces overlapping marginal distributions even when a thermal treatment is applied. The appropriate test is therefore not a comparison of raw temperature distributions but the GAMM model, which explicitly accounts for time-of-day, calendar date, and nested random effects. This analysis confirmed that heat nests were significantly warmer (+2.32 ± 0.55°C, $p<0.001$) and cold nests significantly cooler (-1.31 ± 0.57°C, $p=0.02$) than controls, at least during part of the d2-d8 measured period. During the peak thermal window (12pm-8pm), separation was even more pronounced (heat: +1.94°C, cold: -2.02°C, both $p<0.001$).

To address the reviewer's concern, we performed supplementary analyses using the actual mean nest temperature experienced by nestlings rather than the treatment variable. These analyses gave results qualitatively similar to those presented in the manuscript, with no significant associations between nest temperature and any morphological or physiological trait. This

© 2026. Published by The Company of Biologists under the terms of the Creative Commons Attribution License (https://creativecommons.org/licenses/by/4.0/).

additional analysis suggests that the absence of detectable developmental effects is not simply explained by overlap in temperature distributions among treatment groups. To better emphasize these aspects in the manuscript, the following paragraph is now added to the discussion L. 308-318: “The pronounced daily and seasonal temperature fluctuations we recorded underscore how dynamic the thermal environment of free-living nestlings is, with natural temperature variations frequently exceeding the 1-2 °C differences created here experimentally (e.g., average temperature of the control nestboxes range 13.9-23.1°C, Fig. S1). Such high background variability likely reduced the effective contrast between treatments and may have masked subtle effects. After accounting for temporal variation, heated nests remained significantly warmer and cooled nests significantly cooler than control nests, with treatment differences approaching 2°C during the warmest part of the day. Thus, nestlings experienced distinct thermal environments despite the high variability characteristic of natural nest conditions. Consistent with this interpretation, analyses based on realized nest temperatures rather than treatment identity showed qualitatively similar results (Table S3).”

Reviewer 2: Note on raw data: I did not see raw data appended in the reviewer portal.

Authors: The raw data are now added to Zenodo repository: https://zenodo.org/records/21338492

General comments: In this study, the authors manipulated nest temperature in wild nesting Great Tits to study whether and how heat and cold stress affect development and physiology.

Despite honesty about how modest temperature manipulations were, I would have liked to see the authors put this into more context and better define why these small-scale changes were "ecologically relevant".
Based on what the authors mentioned about this Great Tit population being a high-latitude population accustomed to large temperature fluctuations, the temperature treatments induced (target: +/- 2 degrees C) perhaps too subtle to produce the signal the authors had anticipated.

Authors: We agree that the magnitude of our thermal manipulation was modest, our intention was not to simulate extreme thermal events, but rather to reproduce realistic variation in nest microclimate that nestlings may naturally experience over short temporal scales during the breeding season and to experimentally simulate ecologically realistic temperature deviations (±2°C), consistent with near-term climate projections (IPCC, 2023). Importantly, developmental processes in altricial birds occur within relatively narrow thermal windows, and previous studies have shown that even small changes in developmental temperature can influence survival, growth or physiological mechanisms (Rodríguez and Barba, 2016; Corregidor-Castro and Jones, 2021; Furic et al., 2025). We also chose moderate manipulations deliberately to avoid exposing nestlings to temperatures outside the natural range experienced in nestboxes or nest cavities which help buffer chicks from extreme ambient temperature fluctuations.

We have now clarified in the introduction why we consider these treatments ecologically relevant L. 71-76**:** “The magnitude of our temperature manipulation (±2°C) was chosen to reflect near-term climate change projections (IPCC 2023), representing the chronic thermal deviation nestlings are likely to experience within coming decades, as opposed to the acute extremes that characterize current heatwaves or cold snaps.
While such deviations are modest relative to the natural daily temperature fluctuations, it is precisely this ecologically realistic scale that is most relevant for predicting population-level responses to ongoing climate change.”

Reviewer 2: Various parts of the introduction and discussion were somewhat superficial and would be strengthened by inclusion of relevant work from other wild bird systems. For example, research teams working on Tree Swallows have actively manipulated temperatures in nest boxes for years and have assessed physiological (mostly stress parameters) and developmental impacts on nestlings (e.g., Taff et al. 2026, Current Biology; see other work by Taff and Vitousek). Additionally, several recent studies have looked at longer term impacts of inclement temperature fluctuations on fitness (Taff and Shipley 2023, Nature Communications https://www.nature.com/articles/s41467-023-43071-y), as well as a phenology, morphology, and aspects of reproduction (e.g., Stager et al. 2026, Nature Ecology and Evolution). Finally,

© 2026. Published by The Company of Biologists under the terms of the Creative Commons Attribution License (https://creativecommons.org/licenses/by/4.0/).

theoretical models about resilience and robustness (e.g. Vitousek et al. 2025, Trends in Ecology and Evolution) would provide a conceptual basis for the current work that would better ground the authors' predictions. It would additionally be helpful for the authors to discuss how genetic background could impact scope of response and capacity for developmental plasticity within nestlings.

Authors: We thank the reviewer for this constructive suggestion and have revised the Introduction and Discussion to better situate our work within the broader literature on developmental thermal environments in wild birds. We now include additional empirical and conceptual context from tree swallow studies from Taff et al. (2026) and Taff and Shipley (2023). The following paragraph was added to the Introduction and reads L. 60-68: "studies in wild bird show that these responses are often strongly context-dependent and can vary with environmental conditions, developmental stage, and parental buffering (Taff et al., 2026; Taff and Shipley 2023). Additionally, responses to thermal variation may also depend on population history and local adaptation. In great tits, population differences in reaction norms to environmental conditions have been reported even in the presence of gene flow (Broggi et al. 2005), indicating that phenotypic plasticity can vary across populations. This suggests that responses to thermal variation may not be uniform across the species' range, highlighting the value of quantifying developmental responses in ecological realistic conditions within specific populations."

We complete this point by adding to the discussion the following paragraph L. 398-402: "Additionally, this study was conducted within a single great tit population, and thus captures within-population responses to thermal variation rather than differences in reaction norms among populations. Although population-specific environmental history may shape developmental plasticity in this species, testing such hypotheses requires comparative approaches across multiple populations."

We have also incorporated the study of Taff et al., 2026 into the Discussion to better contextualize the possibility that developmental thermal environments may have delayed or latent effects that are not immediately detectable during the nestling stage L. 389-398: "Our results contribute to a growing body of work on the developmental effects of thermal environments in wild birds (Rodriguez and Barba, 2016a, b; Andreasson et al., 2017; Taff et al. 2026; Taff and Shipley 2023; Satarkar et al., 2026). Experimental studies in other species (e.g. pied flycatchers, ±2°C, Furic et al., 2025; blue tits, +1.5 to +3°C, Rodríguez and Barba 2016, 2019) have reported both immediate and latent effects of early-life thermal conditions, even following relatively small temperature manipulations. Great tits are a widespread generalist species occupying a broad range of climatic conditions, and previous work has shown that thermal reaction norms can vary among populations (Broggi et al. 2005), suggesting that developmental responses may be shaped by local environmental history. Within this context, the absence of short-term effects observed here may reflect population-specific sensitivity to ecologically realistic thermal variation."

Specific comments:

Reviewer 2: Lines 57-59: But see work by Tree Swallow folks, for example. Also see Xue et al. 2025, Nature Communications.

Authors: We have now revised the mentioned section to more explicitly acknowledge that thermal manipulations in wild populations often produce context-dependent physiological and developmental responses L. 56-68: "The physiological mechanisms underlying thermal stress are relatively well characterized, particularly in controlled settings. Heat and cold exposure affect several key biological systems including mitochondrial function, reducing the efficiency of ATP production while often stimulating mitochondrial proliferation (Akbarian et al., 2016), and immune function, frequently leading to a reduction in antibody production and immune cell activity (Siddiqui et al., 2022; Li et al., 2025). However, studies in wild bird systems show that these responses are often strongly context-dependent and can vary with environmental conditions, developmental stage, and parental buffering (Taff et al., 2026; Taff and Shipley 2023). Additionally, responses to thermal variation may also depend on population history and local adaptation. In great tits, population differences in reaction norms to environmental conditions have been reported even in the presence of gene flow (Broggi et al. 2005), indicating that

© 2026. Published by The Company of Biologists under the terms of the Creative Commons Attribution License (https://creativecommons.org/licenses/by/4.0/).

phenotypic plasticity can vary across populations. This suggests that responses to thermal variation may not be uniform across the species' range, highlighting the value of quantifying developmental responses in ecological realistic conditions within specific populations. "

Reviewer 2: Line 72: the phrase "thermoregulatory capacity" should be better defined - what is considered within the 'normal' scope for adult birds, and how does this vary with genetic background and environment?

Authors: Thank you for this suggestion, we have revised the sentence to clarify what is meant by 'thermoregulatory capacity' in this context. The revised sentence now reads L. 83-87: "To our knowledge, relatively few studies have simultaneously assessed both developmental and physiological responses in the wild, and most have focused on temperature manipulations during later nestling stages, when thermoregulatory capacity is already emerging i.e. when nestlings are beginning to achieve endothermic competence and are less dependent on parental brooding and nest microclimate for maintaining their body temperature."

Detailed discussion on the effects of genetic background and environment has been added L. 398-402: "Additionally, this study was conducted within a single great tit population, and thus captures within-population responses to thermal variation rather than differences in reaction norms among populations. Although population-specific environmental history may shape developmental plasticity in this species, testing such hypotheses requires comparative approaches across multiple populations."

Reviewer 2: Lines 73-74: The authors should provide more information on how these rough temperature thresholds were selected. Presumably, 2 degrees C is within the normal range of variation that a nestling might experience when a parent leaves the nest to forage, no? The phrase 'ecologically relevant' therefore seems context dependent. Furthermore, what is relevant to a cavity nester may not be ecologically relevant to an open cup nester in a less protected environment. In short, this small-scale temperature manipulation seems so slight that it would be unlikely to impact nestlings, so some justification for why it was chosen is warranted.

Authors: Our objective was not to expose nestlings to extreme temperatures or to exceed their thermal tolerance limits, but rather to simulate ecologically realistic changes in nest temperature consistent with near-term climate projections (approximately ±2°C; IPCC 2023). We agree that the ecological relevance of such a temperature difference depends on species, nesting ecology, and local environmental conditions, and we have clarified this point in the revised Introduction L. 69-76: "In this study, we experimentally manipulated nest temperature during early postnatal development in wild great tits to test how heat and cold challenges affect nestling growth, survival, and physiological condition. The magnitude of our temperature manipulation (±2°C) was chosen to reflect near-term climate change projections (IPCC 2023), representing the chronic thermal deviation nestlings are likely to experience within coming decades, as opposed to the acute extremes that characterize current heatwaves or cold snaps. While such deviations are modest relative to the natural daily temperature fluctuations, it is precisely this ecologically realistic scale that is most relevant for predicting population-level responses to ongoing climate change."

Additionally, throughout the manuscript, we have replaced the term "thermal stress" with "thermal challenge", which more accurately reflects our experimental design without presupposing a physiological stress response.

Reviewer 2: Lines 86-87: Why did the authors predict that heat- and cold-stressed birds would both suffer reduced body mass?

Authors: We agree that our sentence implied a common energetic mechanism underlying reduced growth in both heated and cooled nests. Our rationale was that warmer or cooler nest temperatures could alter developmental conditions and energy allocation compared to control nests. We have revised the text L. 87-94: "Because maintaining body temperature under either heat or cold challenge during this vulnerable developmental period can divert energy away from growth processes, we predicted that nestlings in both heated and cooled nests would exhibit (i)

© 2026. Published by The Company of Biologists under the terms of the Creative Commons Attribution License (https://creativecommons.org/licenses/by/4.0/).

reduced body mass and wing length compared with controls, (ii) increased mortality risk during the nestling period due to heightened energetic demands, dehydration, or thermoregulatory failure, (iii) signs of dehydration in heat-exposed nestlings (elevated plasma osmolarity and uric acid), (iv) increased mitochondrial density (as a compensatory response to increased energy demand) in both heat and cold groups and (v) reduced plasma immunoglobulin concentrations through stress-induced immune suppression."

Reviewer 2: Lines 104-107: This sentence seems to provide further evidence that increases or decreases in temperature of 2 degrees C may have negligible impacts on nestlings.

Authors: We would like to clarify that the sentence in L 104-107 was not intended to suggest that ±2°C temperature changes have negligible biological effects. Rather, it refers to the ecological relevance of studying nestlings in a high-latitude system where thermal conditions are inherently variable during the breeding season. In such environments, nestlings are regularly exposed to short-term temperature fluctuations. Importantly, even relatively small temperature changes can have biological consequences in early life stages, however, their magnitude and detectability may depend on population-specific reaction norms and the temporal scale of exposure.

We have clarified this point in the revised manuscript to avoid any interpretation that small temperature differences are assumed to be biologically negligible L. 106-110 " In high-latitude populations such as those in Finland, nestlings are naturally exposed to pronounced short-term temperature variability during the breeding season (Nord & Nilsson, 2016), making them a relevant system for studying developmental responses to thermal variation under ecologically realistic conditions in the wild."

Reviewer 2: Lines 108-109: Do females incubate continuously or do they leave to forage?

Authors: In great tits, females do not incubate or brood continuously. During both incubation and the early nestling period, females regularly leave the nest to forage, resulting in natural short-term fluctuations in nest temperature (Álvarez and Barba., 2014). After hatching, females provide brooding care primarily during the first days of the nestling period, but brooding intensity declines as nestlings develop and females increasingly leave the nest to forage. Consequently, nestlings are exposed to variable thermal conditions within the nest environment, which is further influenced by external ambient temperature. We modified the following sentence L. 127-131: "Nest temperature was monitored using thermo-loggers iButton® DS1923 temperature/humidity logger, ± 0.06 °C and 0.04%RH Resolution) placed inside the nest box at the beginning of the treatment (d2), 5 cm above the chicks, measuring temperature every 10min.Therefore, temperature recorded reflects the actual nest microclimate experienced by nestlings, which is shaped by ambient conditions and parental brooding behaviour (Álvarez and Barba, 2014)."

Reviewer 2: Lines 121-122: Abstract lists temperature reduction as approximately -1.3 degrees C; 2 degrees C is listed here.

Authors: The experimental design aimed to manipulate nest temperature by approximately ±2°C but, in practice, the cooling treatment was less effective than expected, resulting in an average reduction of approximately -1.3°C. We have now modified the corresponding sentence that now reads L. 120-125: "Treatment consisted on the installation of two heating pads (AquaPack HeatPaxx 72 h) or cooling pads (VWR 606 g, frozen at -20C) in pouches on the outer lateral sides of the wooden nest boxes,surrounded by insulation, to modify nest temperature. This resulted in an average increase of approximately +2.3°C in heated nests and a decrease of approximately -1.3°C in cooled nests (Stier et al., 2022; Furic et al., 2025)."

Reviewer 2: Lines 124-125: specify the precision of the ibuttons used.

Authors: This information is now added L. 127-128: " Nest temperature was monitored using thermo-loggers (iButton® DS1923 temperature/humidity logger, ± 0.06 °C and 0.04%RH Resolution)"

© 2026. Published by The Company of Biologists under the terms of the Creative Commons Attribution License (https://creativecommons.org/licenses/by/4.0/).

Reviewer 2: Lines 224-233: It would be useful to describe how temperatures inside each nest box varied relative to ambient external temperature (seasonality is a covariate here). Did the authors examine growth and development in relation to the timing of nesting during the season, e.g., early vs late nesters?

Authors: Nest box temperatures were strongly influenced by ambient environmental conditions, as reflected in the significant effects of time of day and calendar date in the GAMM model. The calendar date effect captures seasonal variation in external temperature across the breeding season, which is transmitted into nest boxes, while our treatment effects represent deviations from this background environmental variation induced by heating or cooling devices. Thus, nest temperatures reflect both ambient-driven seasonal variation and experimentally induced differences relative to that baseline. To explicitly address this point, we have calculated the temperature difference between nest box and ambient temperature, this is now detailed L. 205-208: “To evaluate how nest box temperatures varied relative to ambient conditions, we calculated the temperature difference between nest box and concurrent ambient temperature (ΔT = nest box temperature - ambient temperature measured at each forest plot, using USB logger data) and compared ΔT among treatments (Control, Cold, Heat) using the same GAMM structure described above.“. We added the results L. 249-252: “On average, nest box temperature in heat group was 4.5°C (95% CI: 4.4-4.5) above concurrent ambient temperature,Cold-treated nests were 0.5°C (95% CI: 0.4-0.5) above ambient, and Control nests were 1.1 C (95% CI: 1-1.4) relative to ambient, presumably reflecting the basic insulating effect of the nest box itself as well as heat generated by the nestlings and parents.” and L. 242-244: “The significant effect of calendar date reflects seasonal changes in ambient temperature across the breeding season, which are transmitted into nest boxes, while treatment effects represent deviations from this background temporal variation.”

Regarding potential seasonal effects on growth and development, we included scaled hatch date as a covariate in all mixed-effects models, thereby accounting for variation associated with early vs. late nesters. This ensures that treatment effects are estimated independently of seasonal timing.

Reviewer 2: Line 305: Add the word "modest" before "early life thermal manipulation".

Authors: Done L. 324: “Contrary to our predictions, modest early-life thermal manipulation did not significantly affect nestling survival or morphometric traits.”

Reviewer 2: Lines 315-317: Several relevant papers to cite here on the importance of parent quality and social signal on nestling development (e.g., McNew et al. 2024 JEB, Taff et al. 2023 Journal of Experimental Zoology Part A: Ecological and Integrative Physiology - both papers about manipulation of social signal and downstream responses), as well as the effect of perceived predation on nestling development (e.g., McNew et al. 2024, Ecology https://drive.google.com/file/d/1OeOqqtrj_WaWi4GeVHgV9zgPxF1BGGdM/view)

Authors: We thank the reviewer for this helpful suggestion and we have now expanded this section to include additional relevant references L. 334-341: “Moreover, variation in mortality was primarily explained by differences among nests, indicating that factors such as microhabitat, parental quality, or predation may play a stronger role than the relatively modest thermal variation imposed here. This interpretation is consistent with experimental studies showing that stressful factors affecting females, including perceived predation risk and socially mediated signals, can have substantial carry-over effects on nestling growth, physiology, and survival (Coslovsky and Richner, 2011; McNew et al. 2024). Additionally, perceived predator exposure during the nestling period can strongly reduce fledging success (Basso and Richner, 2015; Taff et al. 2023), although effects may vary with ecological context and timing (e.g. lack of effects in pied flycatchers, Morosinotto et al., 2019). ”

Second decision letter

MS ID#: bio.062670R1

© 2026. Published by The Company of Biologists under the terms of the Creative Commons Attribution License (https://creativecommons.org/licenses/by/4.0/).

MS Title: Impacts of early-life thermal challenge on development, survival, and physiology in wild great tits

Authors: Julie Fleitz; Clemence Furic; Charli S. Davies; Jenna Palttala; Valentina Giongo; Loan Hognon-Pelletier; Suvi Ruuskanen; Sophie Reichert

I am happy to tell you that your manuscript has been accepted for publication in Biology Open, pending our standard publication integrity checks. It was accepted on 16th July 2026.

© 2026. Published by The Company of Biologists under the terms of the Creative Commons Attribution License (https://creativecommons.org/licenses/by/4.0/).
